# The antiSMASH database, a comprehensive database of microbial secondary metabolite biosynthetic gene clusters

**DOI:** 10.1093/nar/gkw960

**Published:** 2016-10-24

**Authors:** Kai Blin, Marnix H. Medema, Renzo Kottmann, Sang Yup Lee, Tilmann Weber

**Affiliations:** 1 The Novo Nordisk Foundation Center for Biosustainability, Technical University of Denmark, 2800 Kgs. Lyngby, Denmark; 2 Bioinformatics Group, Wageningen University, 6708 PB Wageningen, The Netherlands; 3 Microbial Genomics and Bioinformatics Research Group, Max Planck Institute for Marine Microbiology, 28359 Bremen, Germany; 4 Metabolic and Biomolecular Engineering National Research Laboratory, Department of Chemical and Biomolecular Engineering (BK21 Plus Program), Korea Advanced Institute of Science and Technology (KAIST), Daejeon 34141, Republic of Korea

## Abstract

Secondary metabolites produced by microorganisms are the main source of bioactive compounds that are in use as antimicrobial and anticancer drugs, fungicides, herbicides and pesticides. In the last decade, the increasing availability of microbial genomes has established genome mining as a very important method for the identification of their biosynthetic gene clusters (BGCs). One of the most popular tools for this task is antiSMASH. However, so far, antiSMASH is limited to *de novo* computing results for user-submitted genomes and only partially connects these with BGCs from other organisms. Therefore, we developed the antiSMASH database, a simple but highly useful new resource to browse antiSMASH-annotated BGCs in the currently 3907 bacterial genomes in the database and perform advanced search queries combining multiple search criteria. antiSMASH-DB is available at http://antismash-db.secondarymetabolites.org/.

## INTRODUCTION

A majority of the clinically used antibiotics, but also many drugs for other indications are derived from natural products produced by plants or microorganisms (1). One of the major innovations in the field of (microbial) natural product research during the last two decades was the complementation of classical isolation and analytical techniques with genome mining approaches that allow the identification and characterization of the biosynthetic pathways for natural products based on (meta-) genome data (2). To make this technology available to a broad range of researchers, several sophisticated software solutions have been developed (3–5). Since its initial release in 2010, antiSMASH (6–8) has made significant impact in the community as one of the most widely used software pipelines for secondary metabolite genome mining.

However, antiSMASH is designed as a genome mining pipeline for analyzing individual genomes and does not provide interconnections or cross-genome search functionality. Therefore, we now have developed the antiSMASH database, which contains pre-calculated antiSMASH results for all publicly available (cut-off date May 27th, 2016) microbial genomes from the NCBI GenBank database that have an assembly status of ‘complete’ and existing gene calls (currently 8883 records of 3907 unique species). At regular time intervals, all entries are re-analyzed with the newest version of antiSMASH. Available genomes can be browsed by taxonomy (based on the NCBI taxonomy annotation) or searched by NCBI accession number, genus, species or strain. Additionally, the interactive web interface assists users in constructing complex queries fulfilling a range of needs: e.g. a synthetic biologist might want to quickly find all BGCs of type ‘non-ribosomal peptide synthase’ that incorporate the non-proteinogenic amino acid dihydroxy-phenylglycine for engineering purposes; or an evolutionary genomicist might want to retrieve ‘all lanthipeptide prepeptide-encoding genes encoded in the genomes of the genus *Streptomyces*’. Identified gene clusters can be viewed online. To support variable downstream workflows, results can also be downloaded as tabular files. Corresponding BGC, gene and NRPS/PKS-domain sequences can be exported as FASTA files.

The antiSMASH database provides researchers with an easy to use, up-to-date collection of state-of the art annotated BGC data, which enable them to easily perform cross-genome analyses by offering complex queries on the data sets. In many aspects, the antiSMASH database covers a wider scope of information than existing databases such as doBISCUIT (9) ClusterMine360 (10) that focus on a limited set of BGCs, StreptomeDB (11,12) only focusing on natural products from *Streptomycetes* and IMG-ABC (13) not annotating secondary metabolite clusters of orthologous groups (smCOGs), active site predictions or the improved RiPP annotations added in the 3.0 release of antiSMASH. Moreover, users benefit from a rich set of contextual data, because of the tight integration with the Minimum Information about a Biosynthetic Gene cluster (MIBiG) repository (14).

## antiSMASH DATABASE ARCHITECTURE

**Figure 1.**
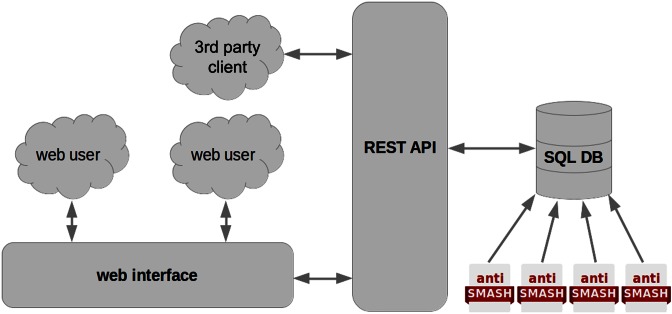
Overview of the modular architecture of the antiSMASH database.

The antiSMASH database is a multilayer web service (Figure 1). The front-end web user interface is an AngularJS (https://angularjs.org/) single-page application talking to a REST-like (15) web service using AJAX calls to exchange data in JSON format. The web service layer is implemented in Python using the Flask framework (http://flask.pocoo.org/) and provides an abstraction layer over the SQL database schema (https://github.com/antismash/db-schema, also see Supplementary Figure S1). The data are stored in a PostgreSQL database (https://www.postgresql.org/). Processed antiSMASH results are stored on disk and linked to from the web application where applicable.

### Selection of included genomes

In order to provide the highest possible quality for the antiSMASH annotations, bacterial genomes were taken from NCBI GenBank (16) only when they were annotated as ‘complete genome’. The GenBank annotation instead of the RefSeq (17) annotation was used to avoid issues in cluster detection that were observed with the gene annotations provided by the NCBI prokaryotic genome annotation pipeline (PGAP) (18) used to do the RefSeq gene calls. The GenBank records instead use gene annotations provided by the researchers who sequenced and uploaded the genome. As the externally provided annotations are of mixed quality, another filter step was added to exclude records that have no gene annotations in GenBank. As a last step, all pairs of genomes that contained duplicate ‘locus tag’ annotations were removed.

### antiSMASH annotations and data import

For all input genomes, antiSMASH analyses were run with the same parameter selection that is being used by the defaults on the antiSMASH website (ClusterBlast, SubClusterBlast, KnownClusterBlast, smCOG analysis and active site finder) and the same 3.0.5 release of antiSMASH. Loading data for profile detection and ClusterBlast was changed to more efficiently support running thousands of genomes in a massively parallel fashion, but the analysis code is unchanged from the released version, giving identical results. From the antiSMASH-annotated GenBank files, the information to populate the database was extracted with a custom python script (https://github.com/antismash/db-import).

### REST-like web service

Built with the Python-based Flask framework (http://flask.pocoo.org/), the web service layer (https://github.com/antismash/db-api) provides a REST-like interface (15) for both the web user interface and potential third-party clients interacting with the antiSMASH database. The web service abstracts away the technical details of dealing with the SQL schema and allows to bundle all SQL logic in one place.

## APPLICATION OF THE antiSMASH DATABASE

**Figure 2.**
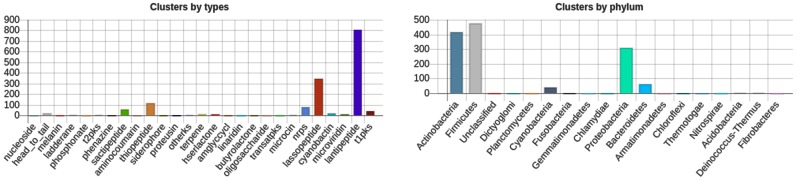
Summary statistics for a search listing all RiPP clusters except for bacteriocins.

The antiSMASH database allows antiSMASH to cross-link many of the ClusterBlast results that pinpoint similar gene clusters, a much requested feature from antiSMASH users. This cross-linking feature is available for results in the database already and will also be included in the next standalone antiSMASH release. The cross-links allow researchers to further investigate similar gene clusters, beyond the gene cluster layout presented directly in the ClusterBlast results. The database also provides summary statistics about secondary metabolite types and taxa. There is a general statistics page giving a high-level overview. Search results also provide summary statistics for the clusters contained in the results (e.g. see Figure 2).

### Example applications for the antiSMASH database queries

In addition to the general statistics on database and the ability to browse the results by secondary metabolite type or taxonomy, it is also possible to perform custom queries. Simple queries work by just entering a search term into the simple search field. Search terms will automatically be matched against BGC types, NCBI accession numbers and genus and species names. If no logical operators are present, multiple terms will be connected using AND logic, so only results that hit against all search terms will be shown. It is also possible to use explicit AND, OR and EXCEPT logic, and order operations using braces.

To more easily build complex queries, the query builder can be used. It makes it easy to build queries for advanced use cases. Using the query builder, search terms can be connected using AND, OR and EXCEPT logic.

### Example of finding all nonribosomal peptide clusters that incorporate dihydroxyphenylglycine

**Figure 3.**
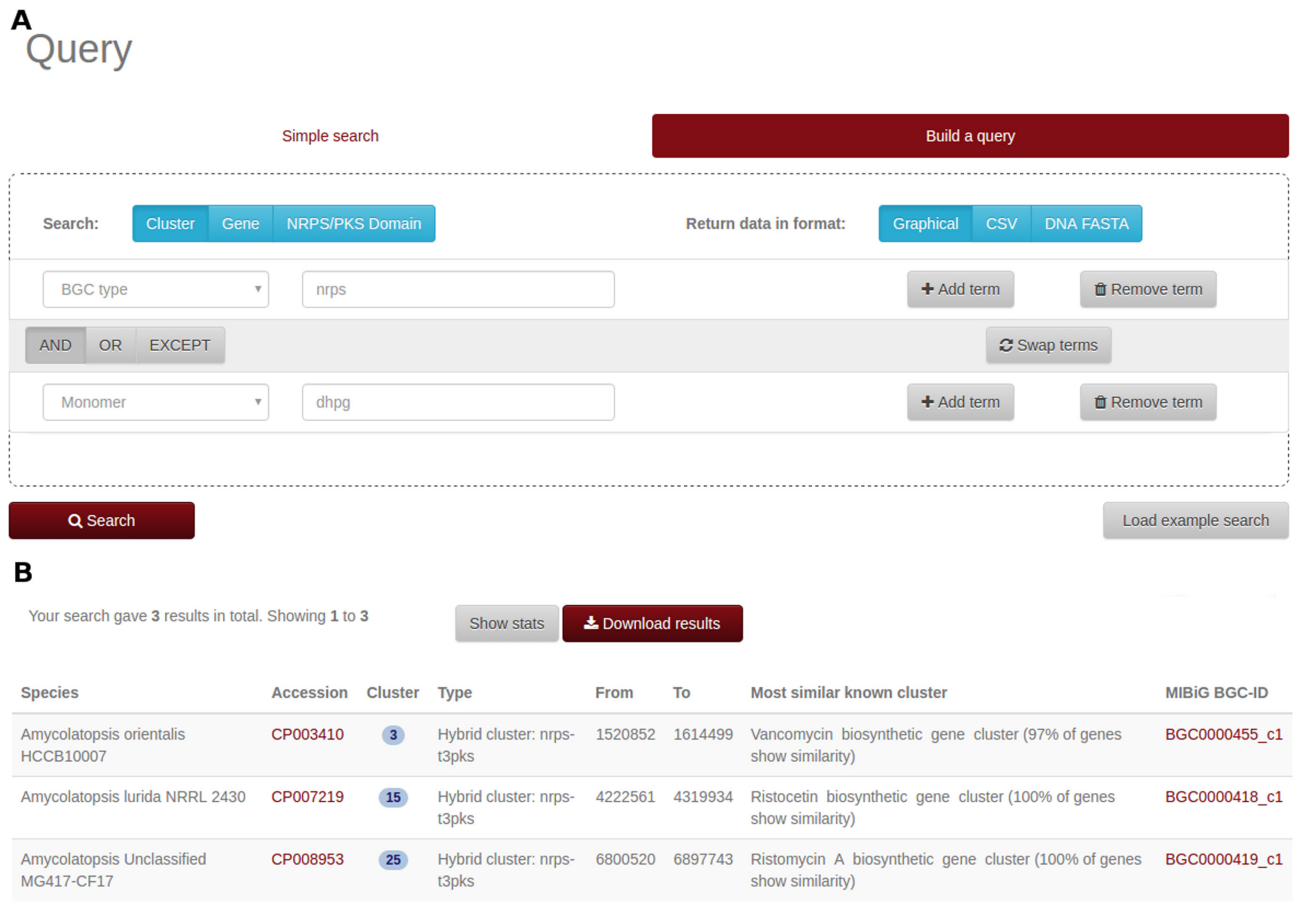
(**A**) Building a query for NRPS clusters incorporating dihydroxyphenylglycine (**B**) Search results for query.

On the ‘Build a query’ tab, a user can click the ‘Add term’ button to create a second search term. For the first term, he or she might, e.g. select ‘BGC type’ as category and enter ‘nrps’ as the search string. For the second term, he or she could, e.g. select ‘monomer’ as category and enter ‘dhpg’ as search string (see Figure 3A), and hit ‘search’.

Currently three BGCs in the database match these search criteria and are returned on the results page (Figure 3B).

### Example of finding all ribosomally synthesized and posttranslationally modified peptide clusters in the Genus *Streptomyces* that are not lantipeptides

**Figure 4.**
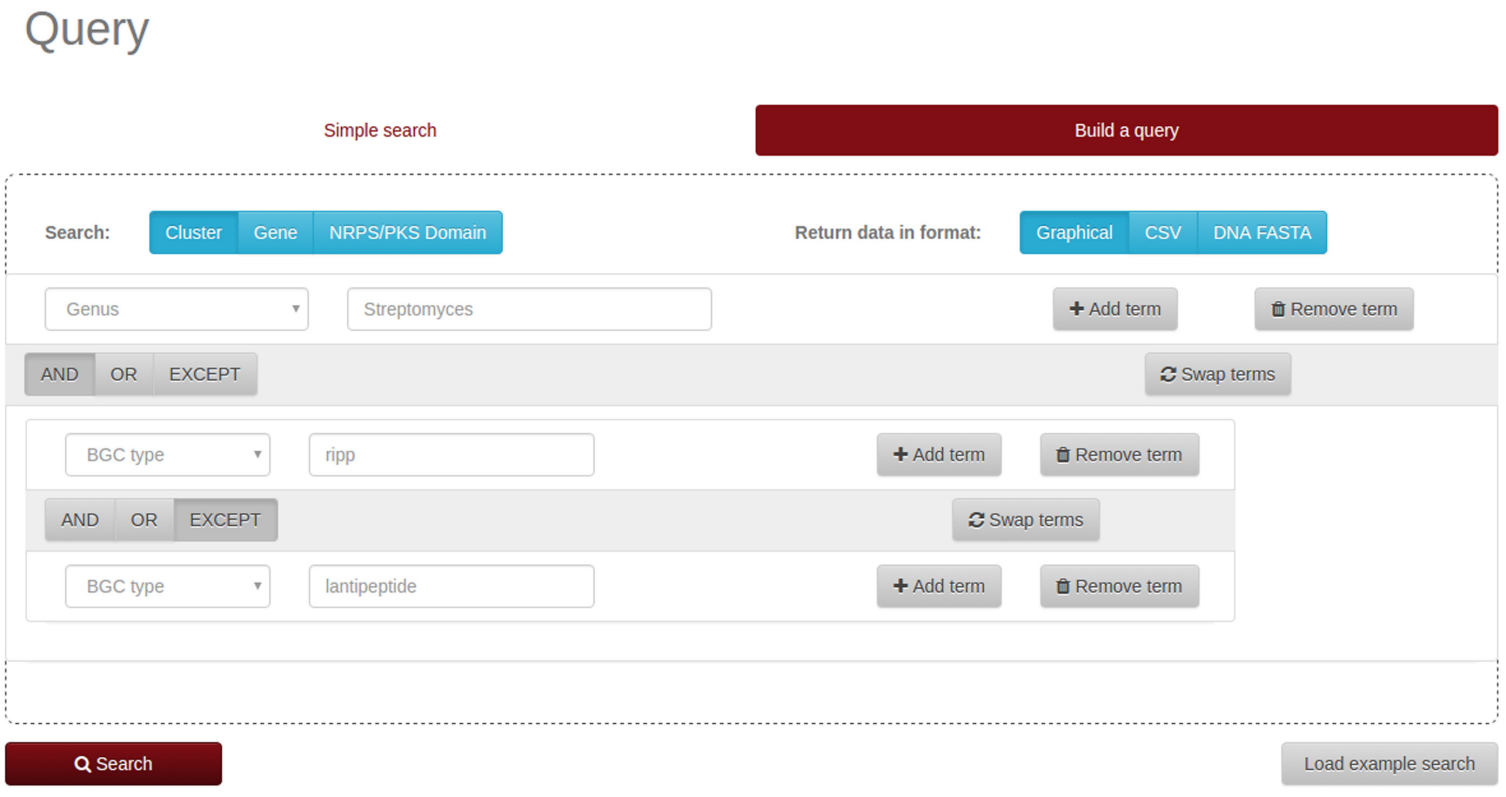
Building a query for all RiPP clusters except lanthipeptides in the Genus *Streptomyces*.

On the ‘Build a query’ tab, a user could click the ‘Add term’ button twice to create a second and third search term. For the first term, he or she could select ‘Genus’ as category and enter ‘*Streptomyces*’ as the search string. For the second term, ‘BGC type’ would be ‘RiPP’. The operation should be switched from ‘AND’ to ‘EXCEPT’. The third term, ‘BGC type’ could be selected as category and ‘lantipeptide’ entered as search string (see Figure 4). This query currently yields 265 clusters. To save the results for further analysis, the result table can be downloaded as CSV file that can then be opened in a spreadsheet application.

Further examples that illustrate how to export gene and NRPS/PKS domain sequences in CSV or FASTA formats can be found on the antiSMASH database website.

## DISCUSSION

To better explore the biosynthetic potential of microorganisms, genome mining is a valuable approach. Since 2011, antiSMASH has been assisting researchers in their secondary metabolite genome mining projects. The antiSMASH website has processed almost 260 000 analysis jobs in the last five years. In order to more quickly access antiSMASH results, to further improve their utility and to better allow researchers to put them into context, we now present the antiSMASH database. We did not use RefSeq annotations as we found that the PGAP annotations used there occasionally missed core biosynthetic genes of secondary metabolite clusters, thus undermining the detection algorithms used by antiSMASH. We are currently working with the NCBI to get these issues resolved and are hoping to be able to switch to the more consistent RefSeq annotations in a future release of the antiSMASH database. To avoid adding partial or fragmented BGCs, genomes that are not assembled to ‘complete genome’ level are not considered at the moment.

### Comparison to related work

There are a number of related databases for researchers interested in microbial natural products. We feel that the antiSMASH database nicely integrates into the existing ecosystem by providing information that is not covered by the established databases. Both doBISCUIT (9) and ClusterMine360 (10) feature manually curated entries describing experimentally characterized BGCs, but are focused on NRPS/PKS clusters. Additionally, the high overhead of manual curation limits the number of available entries. ClusterMine360, the larger of the two databases, currently has 953 entries. Automatic annotation allows the antiSMASH database to drastically increase the coverage: currently, over 9000 NRPS/PKS clusters are available and over 13 000 clusters with other biosynthetic mechanisms. StreptomeDB (11,12) is a very comprehensive database of natural products produced by the genus *Streptomyces*, but does not cover other producers of secondary metabolites. For the genomes covered by the antiSMASH database, *Streptomyces* harbors less than 25% of the clusters from the *Actinobacteria* phylum and only around 6% of the total clusters covered in the database. The IMG-ABC (13) database also provides a large number of automated secondary metabolite annotations, but follows a different philosophy in the annotations. IMG-ABC also utilizes antiSMASH (in the older 2.2 release) to perform cluster predictions, but also enables the probabilistic ClusterFinder (19) provided by antiSMASH. Due to its probabilistic nature, ClusterFinder predicts a large number of ‘putative’ secondary metabolite gene clusters where the biological interpretation of the results is difficult, lowering the confidence in the predictions. Additionally, IMG-ABC includes all genome data available to the JGI, regardless of the quality of the genome assembly or availability to the general public. Based on our extensive experience of running antiSMASH on genome assemblies of different qualities, we know that low quality genome assemblies as input result in low quality metabolite predictions. In the antiSMASH database, we use the latest antiSMASH version to provide profile-based higher confidence predictions on higher quality assemblies, on genomes publicly available from the NCBI GenBank database. Additionally, antiSMASH DB provides a number of further antiSMASH-derived predictions. It features the smCOGs annotations, the active site finder prediction of biosynthetic activity and tight integration into the MIBiG repository (14) for the most similar known gene clusters. All in all, the availability of both these complementary tools now allows users to browse predicted BGCs in both the context of the antiSMASH and JGI-IMG frameworks.

In summary, the antiSMASH database is a comprehensive resource of high quality secondary metabolite cluster predictions. It presents BGC annotations using the native antiSMASH display already familiar to researchers, while closely following the state of the art in microbial secondary metabolite predictions. In addition to being able to browse for secondary metabolite clusters by taxonomy and cluster type, more complex searches can be implemented via the graphical query builder.

## AVAILABILITY

The antiSMASH database is available at http://antismash-db.secondarymetabolites.org/. There are no access restrictions for academic or commercial use of the web server. The source code components and SQL schema for the antiSMASH database are available on GitHub (https://github.com/antismash) under an OSI-approved Open Source license.
